# Multicopper oxidase-1 is required for iron homeostasis in Malpighian tubules of *Helicoverpa armigera*

**DOI:** 10.1038/srep14784

**Published:** 2015-10-06

**Authors:** Xiaoming Liu, Chengxian Sun, Xiaoguang Liu, Xinming Yin, Baohai Wang, Mengfang Du, Shiheng An

**Affiliations:** 1 State key Laboratory of Wheat and Maize Crop Science/College of Plant Protection, Henan Agricultural University, Zhengzhou 450002 P.R. China; 2 Department of Agronomy, Xinyang College of Agriculture and Forestry, Xinyang 464000 P.R. China; 3 Tibet Academy of Agricultural and Animal Husbandry Sciences, Tibet Lhasa 850000 P.R. China

## Abstract

Multicopper oxidases (MCOs) are enzymes that contain 10 conserved histidine residues and 1 cysteine residue. MCO1 has been extensively investigated in the midgut because this MCO is implicated in ascorbate oxidation, iron homeostasis and immune responses. However, information regarding the action of MCO1 in Malpighian tubules is limited. In this study, *Helicoverpa armigera* was used as a model to investigate the function of MCO1 in Malpighian tubules. Sequence analysis results revealed that *HaMCO1* exhibits typical MCO characteristics, with 10 histidine and 1 cysteine residues for copper ion binding. *HaMCO1* was also found to be highly abundant in Malpighian tubules. Temporal expression patterns indicated that *HaMCO1* is mainly expressed during larval molting stages. Hormone treatments [the molting hormone 20-hydroxyecdysone (20E) and juvenile hormone (JH)] revealed that 20E inhibits *HaMCO1* transcript expression via its heterodimer receptor, which consists of ecdysone receptor (EcR) and ultraspiracle (USP), and that JH counteracts the action of 20E to activate *HaMCO1* transcript expression via its intracellular receptor methoprene-tolerant (Met). *HaMCO1* knockdown caused a significant decrease in iron accumulation and also significantly reduced transferrin and ferritin transcript expression. Therefore, *HaMCO1* is coordinately regulated by 20E and JH and is required for iron homeostasis in Malpighian tubules.

Multicopper oxidases (MCOs) are enzymes that catalyze reactions using different substrates, such as polyphenols, aminophenols, phenylendiamines, ferrous ion, copper, ascorbate and bilirubin^1^. Traditionally, MCOs include, but are not limited to, laccase, ferroxidase, ascorbate oxidase and ceruloplasmin^2^. A typical MCO usually contains two highly conserved copper centers with four copper atoms. In an MCO-catalyzed reaction, an electron from a substrate is transferred to a type 1 (T1) copper atom and then to type 2/type 3 (T2/T3) copper centers, where oxygen is reduced to water after the gain of four electrons. The T1 center consists of two histidine and one cysteine residues, whereas the T2/T3 cluster comprises eight histidine residues^3^. Copper-binding sites in T1 and T2/T3 centers, with ten histidine residues and one cysteine residue, are considered to be typical characteristics of MCOs^2^.

As the most extensively investigated MCOs, laccases are widely found in bacteria, fungi, plants, insects and vertebrates^3^. At least two MCOs, namely, MCO1 and MCO2, are found in all known insect genomes, and some genomes contain more than two MCOs; for example, five MCOs are found in the *Anopheles gambiae* genome^4^. MCO2 (synonym laccase2), a type of insect MCO, has been thoroughly investigated. In *Manduca sexta*, Mslac2, a laccase2 (Lac 2) ortholog, is most abundant in the epidermis. Most importantly, Mslac2 is also highly expressed in the pharate pupal stage but is poorly expressed in the early pupal stage. This phenomenon is consistent with the sclerotization process of the cuticle, implicating Mslac2 in cuticle tanning^4^. In *Tribolium castaneum*, RNAi-mediated knockdown of *lac2* leads to tanning failure of larval, pupal, and adult cuticles; indeed, *lac2* has been found to be necessary to facilitate normal cuticle tanning^5^. Similarly, the functional loss of *lac2* in *Apis mellifera* results in structural abnormalities of the exoskeleton^6^. Interestingly, *Lac2* is also essential for cuticle tanning in three stinkbugs^7^. Therefore, *Lac2* plays a conserved role in cuticle tanning.

Although MCO1 belongs to the MCO family, the action of MCO1 differs from that of MCO2. MCO2 is abundantly expressed in the epidermis, whereas MCO1 is highly expressed in the midgut and Malpighian tubules during feeding stages. This difference indicates that MCO1 may be involved in diet detoxification^4^. Furthermore, MCO1 may play different roles in insects, though such details regarding its functions remain unknown. In *Nephotettix cincticeps*, lac1S is exclusively expressed in salivary glands and exhibits laccase activity, indicating that lac1S is involved in the rapid oxidation of phenolic substances^8^. The MCO1 ortholog in *Drosophila melanogaster*, CG3759, is up-regulated upon septic injury^9^, and in *A. gambiae* and *M. sexta*, MCO1 is also rapidly up-regulated in response to bacterial injection^10^. These results suggest that MCO1 might participate in the insect immune response. A subsequent study found that *D*. *melanogaster* MCO1 is a functional ferroxidase, with RNAi-mediated knockdown of MCO1 causing a decrease in the level of iron accumulation in the midgut^11^. Furthermore, MCO1 in *D*. *melanogaster* exhibits significant ascorbate oxidase activity, showing that MCO1 more efficiently oxidases ascorbate; as a result, redox systems become altered, influencing various cell signaling pathways^10^. Therefore, MCO1 in insects is likely involved in diverse functions, including monolignol detoxification, immune response, metal metabolism and redox reaction.

In insects, MCO1 is abundant in the midgut and Malpighian tubules. However, its role in Malpighian tubules remains unknown. In this study, *Helicoverpa armigera* (Hübner) was used as a model to investigate MCO1 action in Malpighian tubules. Our results showed that MCO1 is coordinately regulated by two classical hormones, namely, 20-hydroxyecdyson (20E) and juvenile hormone (JH). We also found that MCO1 is necessary to facilitate iron homeostasis in the Malpighian tubules of *H. armigera*. This study provides insight into the hormone-regulated action of MCO1 in *H. armigera*.

## Results

### Sequence analysis of *H. armigera* MCO1

**Figure 1:**
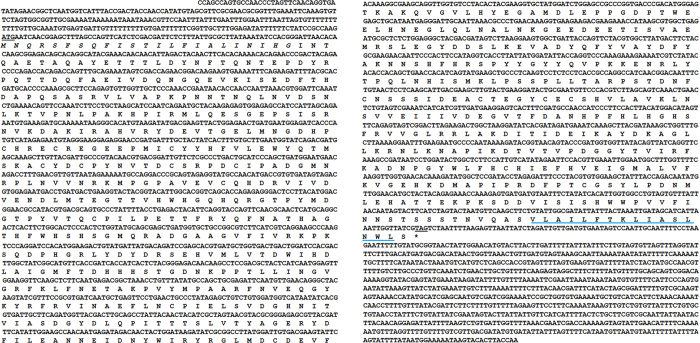
Nucleotide sequence and putative amino acid sequence of *HaMCO1*. The underlining indicates the initiation codon or stop codon. The predicted signal peptide is indicated in italicized text. The putative carboxyl-terminal transmembrane region is delineated with a blue line.

**Figure 2:**
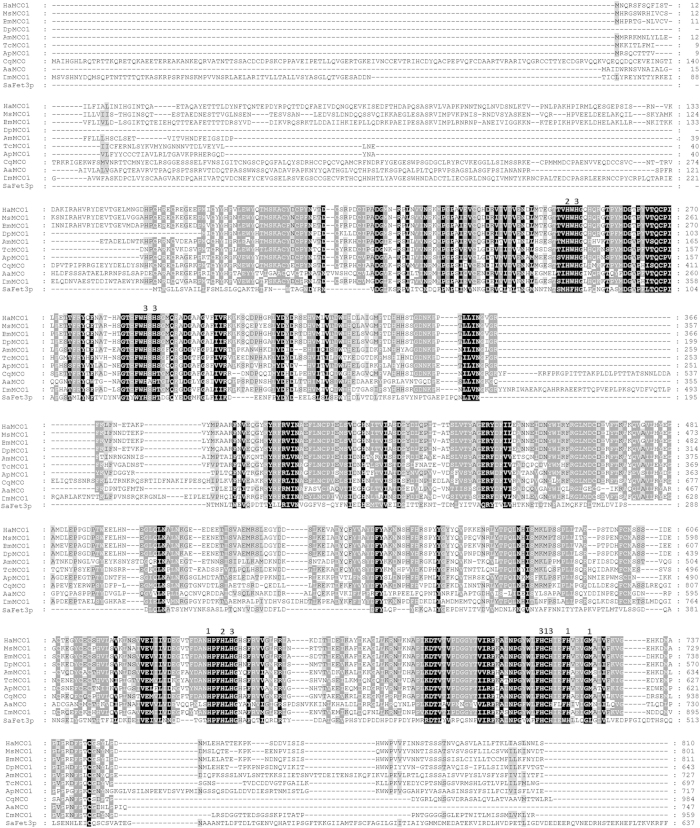
Multiple sequence alignments of the deduced amino acid sequences of *HaMCO1*, *MsMCO1*, *BmMCO1 DpMCO1, AmMCO1, TcMCO1, ApMCO1, CqMCO, AaMCO1, DmMCO1* and *SaFet3p*. The numbering on the right represents the position of the last amino acid in that line. A black box indicates 100% identity, and a grey box presents 100% similarity. The numbers 1, 2 and 3 located above the sequence represent the amino acids involved in coordinating the T1, T2 and T3 copper centers. The sequences (with GenBank accession numbers) for the alignment analysis were as follows: *Helicoverpa armigera*: HaMCO1 (KP318028); *Manduca sexta*: MsMCO1(AAN17506.1); *Bombyx mori*: BmMCO1 (XP_004933574.1); *Danaus plexippus*: DpMCO1 (EHJ67706.1); *Apis mellifera*: AmMCO1 (XP_001120790.2); *Tribolium castaneum*: TcMCO1 (NP_001034514.1); *Acyrthosiphon pisum*: ApMCO1(XP_001948070.1); *Culex quinquefasciatus*: CqMCO (XP_001862911.1); *Aedes aegypti*: AaMCO (AAY29698.1); *Drosophila melanogaster*: DmMCO1 (NP_609287.3); *Saccharomyces arboricola*: SaFet3p (XP_011104804.1).

**Figure 3:**
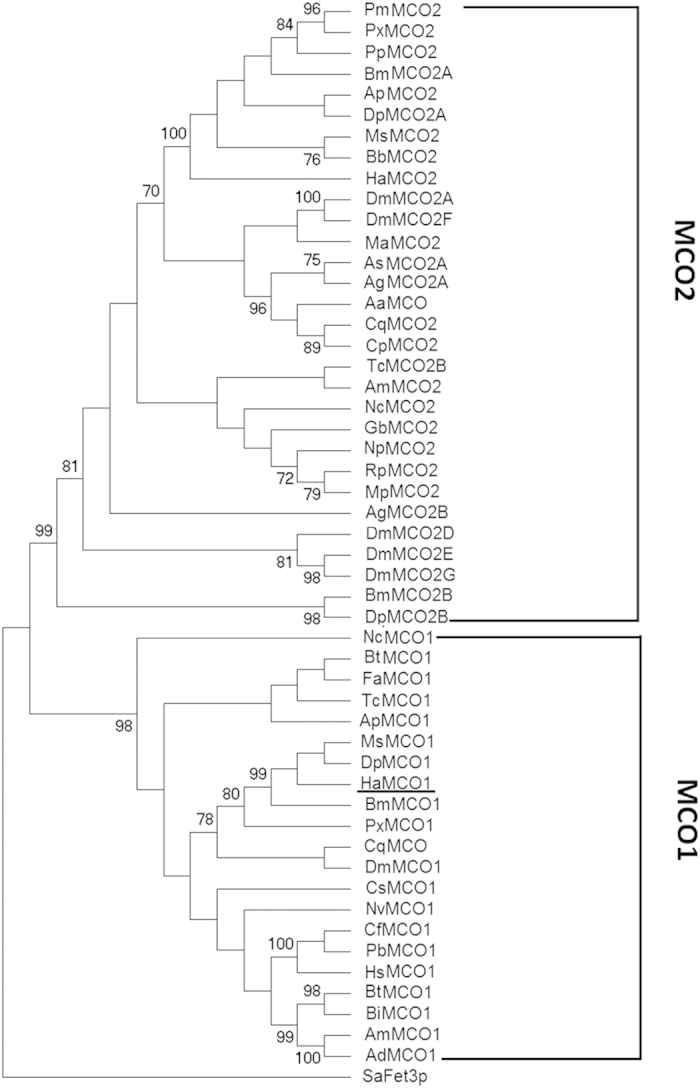
Phylogenetic tree of insect MCO proteins. The phylogenetic tree was constructed using the MEGA4 program (Tamura *et al.*, 2007) according to the neighbor-joining method with a Poisson correction model. The sequences (with GenBank accession numbers) for the phylogenic analysis were as follows: *Helicoverpa armigera*: HaMCO1 (KP318028); *Manduca sexta*: MsMCO1(AAN17506.1); *Bombyx mori*: BmMCO1 (XP_004933574.1); *Danaus plexippus*: DpMCO1 (EHJ67706.1); *Plutella xylostella*: PxMCO1 (XP_011552373.1); *Bombus terrestri*s: BtMCO1 (XP_003394771.1); *Bombus impatiens*: BiMCO1 (XP_003485633.1); *Apis mellifera*: AmMCO1 (XP_001120790.2); *Apis dorsata:* AdMCO1 (XP_006611755.1); *Harpegnathos saltator*: HsMCO1 (XP_011135308.1); *Tribolium castaneum*: TcMCO1 (NP_001034514.1); *Camponotus floridanus*: CfMCO1 (XP_011255366.1); *Acyrthosiphon pisum*: ApMCO1(XP_001948070.1); *Pogonomyrmex barbatus*: PbMCO1 (XP_011645337.1); *Bemisia tabaci*: BtMCO1 (AGC83693.1); *Culex quinquefasciatus*: CqMCO (XP_001862911.1); *Aedes aegypti*: AaMCO (AAY29698.1); *Culex quinquefasciatus*: CqMCO2 (XP_001867157.1); *Culex pipiens pallens*: CpMCO2 (ACG63789.1); *Drosophila melanogaster*: DmMCO1 (NP_609287.3); *Ceratosolen solmsi marchali*: CsMCO1 (XP_011500453.1); *Fopius arisanus*: FaMCO1 (XP_011300938.1); *Nephotettix cincticeps*: NcMCO1(BAJ06131.1); *Nasonia vitripennis*: NvMCO1 (XP_008214237.1); *Helicoverpa armigera*: HaMCO2 (AHA15412.1); *Bombyx mori*: BmMCO2A (BAG70891.1); *Antheraea pernyi*: ApMCO2 (AII19522.1), *Manduca sexta*: MsMCO2 (AAN17507.1); *Danaus plexippus*: DpMCO2A (EHJ72220.1); *Papilio machaon*: PmMCO2(BAJ07600.1); *Papilio xuthus*: PxMCO2 (BAI87829.1); *Papilio polytes*: PpMCO2(BAJ07602.1); *Bombyx mori*: BmMCO2B (DAA06287.1); *Biston betularia*: BbMCO2 (AEP43806.1); *Anopheles sinensis*: AsMCO2A (KFB50921.1); *Anopheles gambiae*: AgMCO2A (AAX49501.); *Drosophila melanogaster*: DmMCO2A (NP_724412.1); *Drosophila melanogaster*: DmMCO2F (NP_724413.2); *Anopheles gambiae*: AgMCO2B (AAX49502.1); *Drosophila melanogaster*: DmMCO2D (NP_001137606.1); *Drosophila melanogaster*: DmMCO2E (NP_610170.2); *Drosophila melanogaster*: DmMCO2G (NP_001260709.1); *Tribolium castaneum*: TcMCO2B (AAX84203.2); *Monochamus alternatus*: MaMCO2 (ABU68466.1); *Riptortus pedestris*: RpMCO2 (BAJ83487.1); *Nephotettix cincticeps*: NcMCO2 (BAJ06133.1); *Megacopta punctatissima*: MpMCO2 (BAJ83488.1); *Gryllus bimaculatus*: GbMCO2 (BAM09185.1); *Apis mellifera*: AmMCO2 (ACK57559.2); *Nysius plebeius*: NpMCO2 (BAJ83489.1); *Danaus plexippus*: DpMCO2B (EHJ72219.1); *Saccharomyces arboricola*: SaFet3p (XP_011104804.1).

The MCO1 sequence designated HaMCO1 was obtained from transcriptome data of *H. armigera* (data not shown). *HaMCO1* contains an open reading frame (ORF) of 2,430 bp, which encodes a putative protein of 810 amino acid residues, with a molecular weight with 91.998 kDa and an isoelectric point of 5.46 (Fig. 1). Similar to MCO1 in other insects, HaCOM1 consists of a secretion signal peptide sequence (located at amino acids 1–23 of the HaMCO1 primary sequence), and a carboxyl-terminal transmembrane region (located at amino acids 794–809 of HaMCO1) (Fig. 1); HaMCO1 is predicted to be GPI-anchored. Most importantly, HaMCO1 also contains ten histidines and one cysteine, which are typical characteristics of MCOs; these residues are required for copper ion binding (Fig. 2). The HaMCO1 amino acid sequence was subjected to further multiple sequence alignments with homologous proteins. The results also revealed that HaMCO1 contains ten histidine residues and one cysteine residue (Fig. 2). Therefore, the sequence obtained from the transcriptome data corresponds to an MCO. Homology analysis showed that HaMCO1 shares 84% amino acid sequence identity with *Danaus plexippus* laccase1, 79% amino acid sequence identity with *Bombyx mori* MCO1, and 75% amino acid sequence identity with *M. sexta* MCO1. Indeed, our obtained *HaMCO* encodes an MCO1. Phylogenetic analysis also revealed that HaMCO1 clusters with the MCO1 of other insects (Fig. 3); hence, the obtained *HaMCO1* sequence is reliable.

### Developmental analysis and tissue distribution of *HaMCO1*

**Figure 4:**
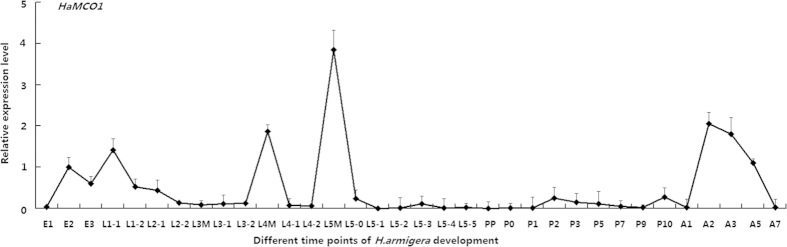
Temporal expression profile of *HaMCO1*. E1, day 1 egg; E2, day 2 egg; E3, day 3 egg; L1-1, day 1 of 1st-instar larva; L1-2, day 2 of 1st-instar larva; L3M, molting stage of 3rd-instar larva; L3-1, day 1 of 3rd-instar larva; L3-2, day 2 of 3rd-instar larva; L4M, molting stage of 4th-instar larva; L4-1, day 1 of 4th-instar larva; L4-2, day 2 of 4th-instar larva; L5M, molting stage of 5th-instar larva; L5-0, 0 h of 5th-instar larva; L5-1, day 1 of 5th-instar larva; L5-2, day 2 of 5th-instar larva; L5-3, day 3 of 5th-instar larva; L5-4, day 4 of 5th-instar larva; L5-5, day 5 of 5th-instar larva; PP, prepupa; P0, 0 h pupa; P1, day 1 of pupa; P2, day 2 of pupa; P3, 3 day of pupa; P5, 5 day of pupa; P7, 7 day of pupa; P9, 9 day of pupa; A1, day 1 of adult; A2, day 2 of adult; A3, day 3 of adult; A5, day 5 of adult; A7, day 7 of adult.

qPCR was performed to investigate the developmental expression pattern of *HaMCO1*. *HaMCO1* was found to be ubiquitously expressed in whole developmental stages. However, *HaMCO1* was much more abundant in the molting stages of fourth- and fifth-instar larvae than in other stages (Fig. 4). Therefore, *HaMCO1* transcript expression is probably associated with 20E and JH.

**Figure 5:**
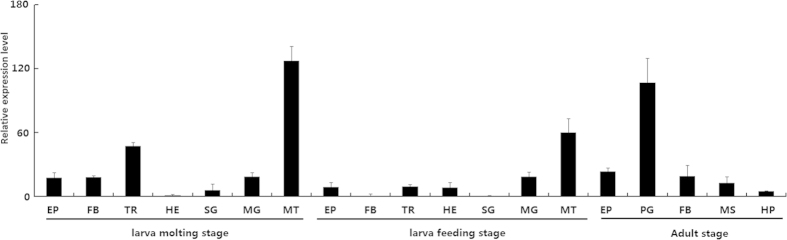
Tissue distributions of *HaMCO1*. EP: epidermis; MD: midgut; FB: fat body; TR: trachea; HE: hemocyte; SG: salivary gland; MT: Malpighian tubule; PG: pheromone gland; MS: muscle; HP: hairpencil.

The tissue distribution of *HaMCO1* was investigated via qPCR. The results revealed that *HaMCO1* transcripts are most abundantly expressed in Malpighian tubules in all the tissues tested at different stages (Fig. 5).

### 20E and JH regulation of *HaMCO1*

**Figure 6:**
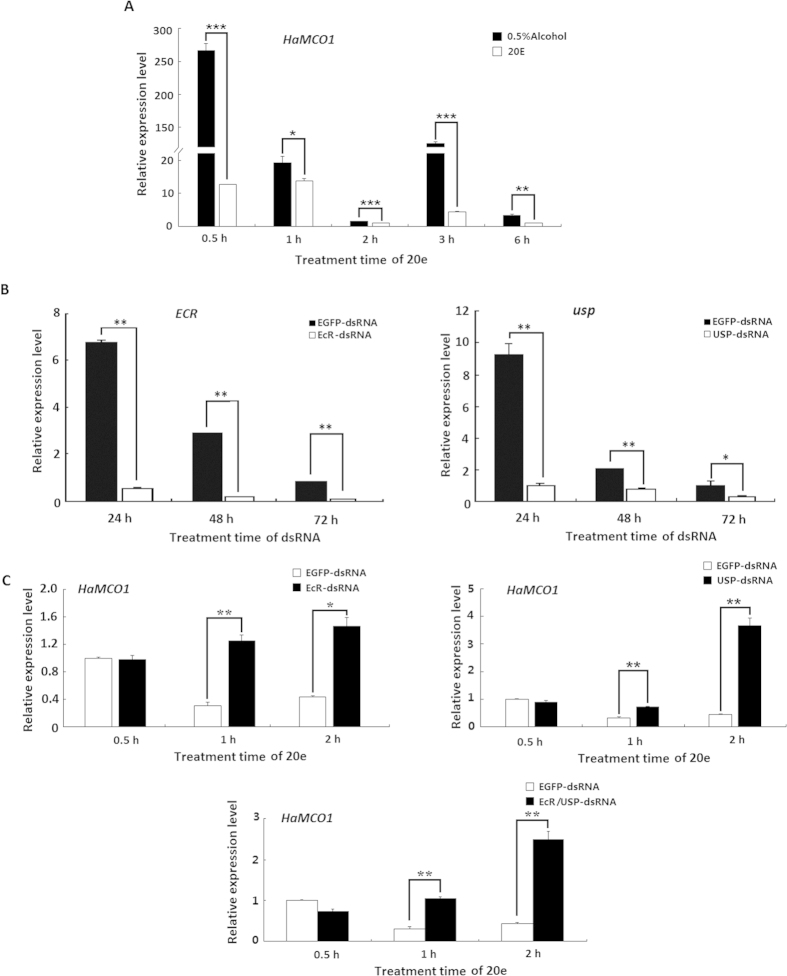
Regulation by 20E of *HaMCO1* transcript expression. (**A**) The effect of 20E treatment on the expression of *HaMCO1* transcript. (**B**) qPCR analysis of *ECR* and *USP* dsRNAi efficiency on *ECR* and *USP* transcript levels, respectively. (**C**) The effect of *ECR* and *USP* knockdown on *HaMCO1* transcript level. The 18S rRNA was used as the housekeeping gene for normalization in all qPCR analyses. The data represent the mean ± SD of three biological replicates. The significance of comparisons were determined by Student’s *t*-test (*p < 0.05, **p < 0.01, ***p < 0.001).

Considering the observed developmental expression patterns, we investigated the effects of two hormones on *HaMCO1* transcript expression. The results showed that *HaMCO1* transcript expression was significantly inhibited after treatment with 20E. The inhibitory effect was rapid: 20E treatments for 0.5 h significantly inhibited *HaMCO1* transcript expression. This inhibitory effect continued until 6 h after 20E treatment was administered (Fig. 6A). The results suggested that 20E inhibits *HaMCO1* transcript expression. 20E-triggered cascades of insect molting and metamorphosis are mediated by its heterodimer receptor, which consists of ecdysone receptor (EcR) and ultraspiracle (USP). Furthermore, RNAi-mediated knockdown of *USP* via injection of *USP* dsRNA resulted in a significant decrease in *USP* mRNA at 24, 48 and 72 h (Fig. 6B). Similarly, RNAi-mediated knockdown of *ECR* also caused a significant reduction in the *ECR* mRNA level at 24, 48 and 72 h after *ECR* dsRNA was injected (Fig. 6B). After 20E receptor (*ECR* or *USP*) expression was successfully inhibited, 20E treatments significantly increased the expression of *HaMCO1* mRNA in comparison with *EGFP* dsRNA treatments (Fig. 6C). These results confirmed that 20E inhibits *HaMCO1* transcript expression via its heterodimer receptor, specifically USP and ECR.

**Figure 7:**
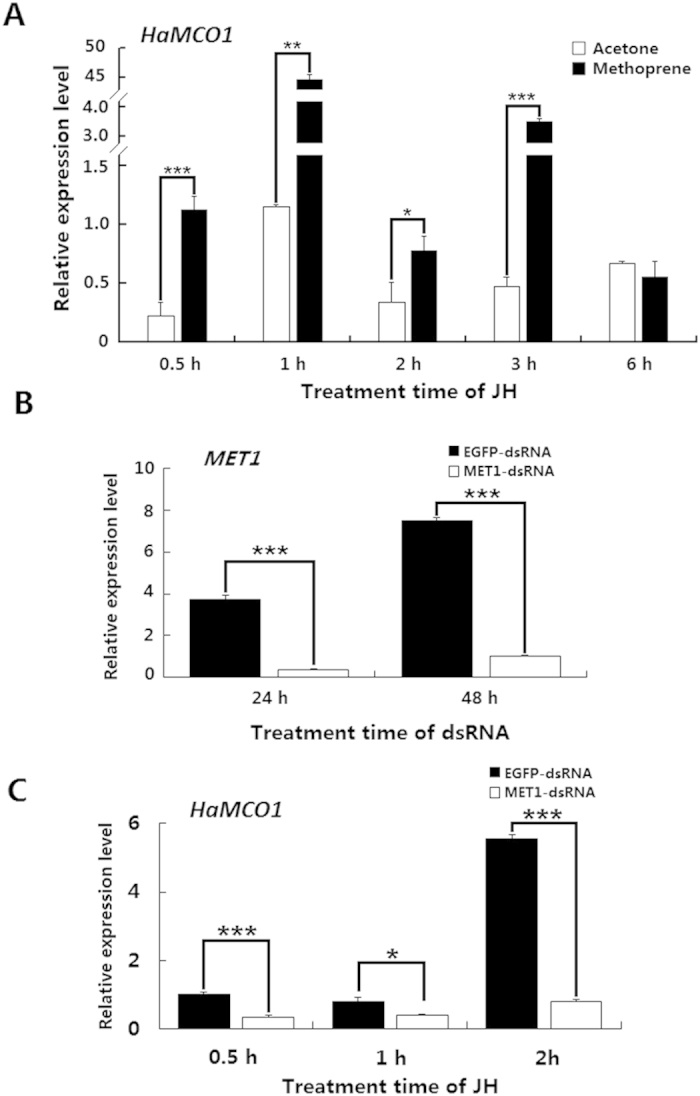
Regulation by JH of *HaMCO1* transcript expression. (**A**) The effect of JH treatment on the expression of *HaMCO1* transcript. (**B**) qPCR analysis of *MET* dsRNAi efficiency on the *MET* transcript level. (**C**) The effect of *MET* knockdown on *HaMCO1* transcript levels. The 18S rRNA was used as the housekeeping gene for normalization in all qPCR analyses. The data represent the mean ± SD of three biological replicates. The significance of comparisons were determined by Student’s *t*-test (*p < 0.05, **p < 0.01, ***p < 0.001).

Considering that 20E inhibits *HaMCO1* transcript expression, we then assessed the role of JH in *HaMCO1* transcript expression. The results revealed that JH treatment caused a significant increase in *HaMCO1* transcript expression (Fig. 7A), indicating that JH activates *HaMCO1* transcript expression. JH also regulated down-stream signals via its intracellular receptor methoprene-tolerant (Met). RNAi-mediated knockdown of *Met1* caused a decrease in the *HaMCO1* transcript level compared with the *EGFP* controls (Fig. 7B). After *Met1* transcript expression was successfully knocked down by RNAi, JH treatment caused a significant decrease in *HaMCO1* transcript levels in *Met1* dsRNAi-treated larvae compared with the *EGFP* controls (Fig. 7C). These results demonstrated that JH promotes *HaMCO1* transcript expression via *Met1* to attenuate the effect of 20E on *HaMCO1* transcript expression.

### *HaMCO1* is required for iron homeostasis

**Figure 8:**
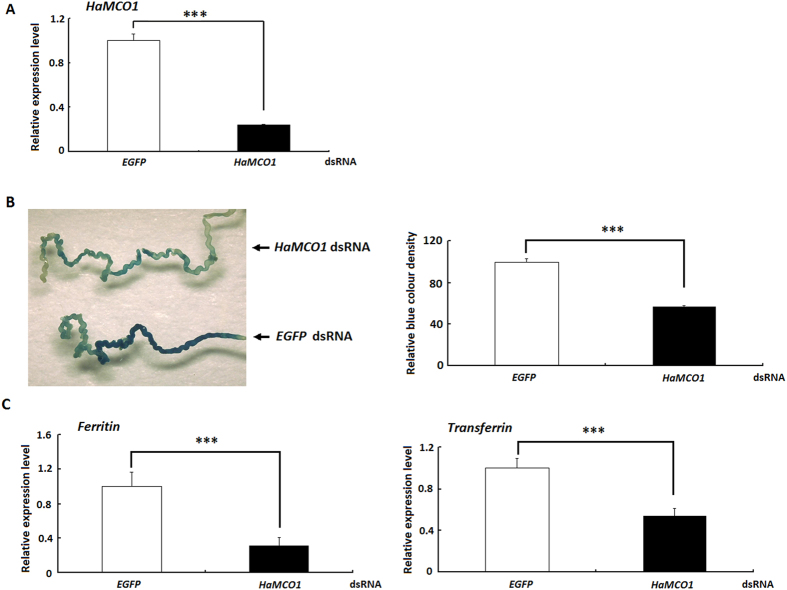
Effect of HaMCO1 knockdown on iron accumulation and the expression of transferrin and ferritin transcripts. (**A**) qPCR analysis of *HaMCO1* dsRNAi efficiency on the *HaMCO1* transcript level. The 18S rRNA was used as the housekeeping gene for normalization in all qPCR analyses. The data represent the mean ± SD of three biological replicates. The significance of comparisons are marked with ***(*p *< 0.001), as determined by Student’s *t*-test. (**B**) The effect of *HaMCO1* knockdown on iron accumulation. (**B-1**) Larval Malpighian tubules from the control (*EGFP* dsRNA) and treatment (*HaMCO1* dsRNA) were analyzed. (**B-2**) The relative blue color density of the control (*EGFP* dsRNA) and treatment (*HaMCO1* dsRNA). The relative blue color density was measured using Quantity One software. The data represent the mean ± SD of three biological replicates. Statistically significant differences were assessed by Student’s *t*-test (***p < 0.001). (**C**) The effect of *HaMCO1* knockdown on the expression of transferrin and ferritin transcripts. qPCR analysis of the effects of *HaMCO1* dsRNA injection on transferrin and ferritin transcript levels. The 18S rRNA was used as the housekeeping gene for normalization in all qPCR analyses. The data represent the mean ± SD of three biological replicates. The significance of comparisons are marked with ***(*p *< 0.001), as determined by Student’s *t*-test.

*HaMCO1* is most abundantly expressed in Malpighian tubules, an important tissue that regulates the balance of water and ions. Accordingly, the effect of *HaMCO1* knockdown on iron homeostasis was assessed. The results revealed that *HaMCO1* knockdown caused a significant reduction in iron accumulation in Malpighian tubules compared with the *EGFP* controls (Fig. 8A,B).

### Effects of *HaMCO1* knockdown on transferrin and ferritin transcript levels

As the knockdown of *HaMCO1* is associated with iron homeostasis, the transcript levels of transferrin and ferritin, which are the key proteins for iron transportation and storage, were assessed in Malpighian tubules of insects with knocked down *HaMCO1*. *HaMCO1* knockdown resulted in a significant decrease in the transcript levels of both transferrin and ferritin (Fig. 8C).

## Discussion

MCOs have similar structures but play different roles based on their substrate specificity. A member of the largest subgroup of MCOs, laccase was first discovered in the Japanese lacquer tree, *Rhus vernicifera*^12^. Since then, laccases have been extensively investigated in plants, fungi and bacteria, and the corresponding structure and functions have also been described in detail^13^. However, insect MCOs have rarely been investigated. In 2004, a laccase gene was first obtained from *M. sexta* and *A. gambiae*^4^. Subsequently, two main forms of MCO were identified in insects: MCO1 and MCO2. MCO2 (laccase2) has been explored in detail, particularly with regard to the conserved role of MCO2 in cuticle tanning in *M. sexta*, *T. castaneum* and *A. mellifera* as well as stinkbugs^4^,^5^,^6^,^7^. However, MCO1 (laccase1) is more complex than MCO2, and the former is also involved in more functions than the latter, including diet detoxification^4^, phenolic substance oxidation^8^, insect immune response^9^, ferrous ion ferroxidase oxidization^11^ and ascorbate oxidization^10^. In the present study, the nucleotide sequence of *HaMCO1* was obtained from transcriptome data and confirmed by PCR (data not shown). Sequence analysis revealed that HaMCO1 contains ten histidines and one cysteine, typical MCO characteristics. Multiple sequence alignments and subsequent phylogenetic analysis further confirmed that the obtained gene encodes MCO1.

Although MCO1 can be detected in the epidermis and fat body in *M. Sexta*, its transcript is also highly expressed in the midgut and Malpighian tubules; similar results have been observed in *A. gambiae*^4^. In the present study, the highly sensitive technique qPCR was employ to compare *HaMCO1* distribution in tissues. The results revealed that *HaMCO1* was most abundantly expressed in Malpighian tubules; *HaMCO1* was expressed to a relatively lower extent in the midgut, indicating that *HaMCO1* most likely plays a more important role in Malpighian tubules. A similar finding was reported for *N. cincticeps*, whereby the *MCO1* gene is exclusively expressed in the salivary glands^8^. These results also confirmed that *MCO1* is involved in diverse functions in insects.

The developmental expression pattern revealed *HaMCO1* to be more abundant in larval molting stages. Considering this finding, we investigated the effect of 20E and JH on *HaMCO1* transcript expression. 20E triggers the molting and metamorphosis of insects at specific times during the life cycle via EcR and USP^14^, and JH coordinates with 20E to regulate molting and metamorphosis. 20E triggers molting, and JH determines the results of molting via the Met receptor^14^. Our results revealed that 20E inhibited *HaMCO1* transcript expression via EcR and the USP receptor. In contrast, JH promoted *HaMCO1* transcript expression via *Met1* to counteract 20E action. Thus, 20E and JH coordinately regulate *HaMCO1* transcript expression, an expression pattern that is different from that of *HaMCO2*: *MCO2* in *H. armigera* is up-regulated by 20E but down-regulated by JH^15^. Similar results have also been found in *M. sexta* and *T. castaneum*; in these insects, the abundance of *MCO2* expression is consistent with the 20E titer^4^,^5^. The expression patterns of *MCO1* and *MCO2* are most likely associated with their corresponding physiological functions. Apolysis is triggered by an increase in the 20E titer, resulting in new cuticle formation and subsequent tanning, and MCO2 must be abundantly expressed after 20E is altered to satisfy the requirements of cuticle tanning. In the apolysis stage, larvae remain immobile and do not ingest food, and corresponding tissue cells undergo rapid programmed cell death (PCD) and a subsequent remodeling process^16^,^17^. As a result, the Malpighian tubules do not function as itself action because of cellular PCD and subsequent tissue remodeling when the 20E titer is high^16^,^17^; thus, 20E most likely inhibits *HaMCO1* transcript expression in Malpighian tubules. A subsequent increase in JH titer induces a larval–larval transition to prevent the larval–pupal–adult metamorphosis triggered by 20E. JH weakens the effect of 20E to promote the larval–larval transition; thus, newly formed larvae must feed to satisfy growth requirements. The corresponding digestive system is activated, and *HaMCO1* functions in Malpighian tubules. Similar results have been reported in *M. sexta* and *A. gambiae*^4^. Surprisingly, although *HaMCO1* transcript expression is regulated by 20E and JH, *HaMCO1* transcript expression was different in most molting stages: high during the molting stages of fourth- and fifth-instar larvae but not in third-instar larvae. The possible reasons are that the 20E and JH titers and the activities of degradation enzymes are different during these stages^18^. For example, it is well known that ECR, the 20E receptor, is up-regulated by higher 20E titer in many insects, though it is down-regulated by a higher 20E titer and up-regulated by a lower 20E titer in *Apis mellifera*^19^. Similar results were found in *Anopheles gambiae*, in which MCO1 is found in relatively low abundance in early-instar larvae and in high abundance in late-instar larvae^20^. However, further study is needed to elucidate this.

Considering that *HaMCO1* is highly expressed in Malpighian tubules, we then investigated the possible role of *HaMCO1* in these structures. RNAi-mediated knockdown of *HaMCO1* resulted in decreased iron accumulation compared with the controls injected with *dsEGFP* RNA. Similarly, a recombinant MCO1 protein in *D. melanogaster* does not exhibit laccase activity *in vivo* but does show ferroxidase activity, a finding that suggests that the MCO1 protein most likely functions as a ferroxidase. The knockdown of *MCO1* in *D. melanogaster* causes a significant decrease in iron accumulation in the midgut^11^. The same results are found in *A. gambiae*, in which *MCO1* knockdown leads to a reduction in iron accumulation^10^. As our study revealed that *HaMCO1* was more abundant in Malpighian tubules, our functional study of *HaMCO1* focused on Malpighian tubules and not on the midgut. Our results implicated *HaMCO1* in iron homeostasis in Malpighian tubules, consistent with the actions of *DmMCO1* and *AgMCO1* in the midgut.

Considering that *HaMCO1* is required for iron homeostasis in Malpighian tubules, we investigated an iron transporter protein (transferrin) and a storage protein (ferritin) in *HaMCO1*-knockdown larvae. The results confirmed that *HaMCO1* knockdown decreased the abundance of transferrin and ferritin transcripts in Malpighian tubules. Because *HaMCO1* exhibits ferroxidase activity, *HaMCO1* knockdown possibly increased the amount of ferrous ions. This increase might act as a negative feedback mechanism to inhibit iron transport and storage; as a consequence, transferrin and ferritin transcript expression could be inhibited. In *D. melanogaster*, MCO1 orthologs demonstrate low ferroxidase and laccase activities but significantly high ascorbate oxidase activity; thus, MCO1 orthologs in insects can possibly function as ascorbate oxidases^10^. In this case, the decreased rate of iron accumulation caused by *MCO1* knockdown possibly contributed to an unknown mechanism. This result is consistent with that a recent study^10^ that suggested that iron homeostasis disrupted by *MCO1* knockdown can be attributed to the disturbance in ascorbate metabolism or the ascorbate redox state^10^. Further studies should be conducted to determine the detailed mechanism of iron homeostasis that are disturbed by *MCO1* knockdown.

## Methods

### Insects

*H. armigera* larvae were reared on an artificial diet at 26 °C under a 16 h light/8 h dark cycle. *H. armigera* adults were fed 10% sucrose.

### Sequence analysis

The *H. armigera* MCO1 nucleotide sequence was obtained from *H. armigera* transcriptome data. The nucleotide sequence was further confirmed using PCR (data not shown). The putative signal peptide of the amino acid sequence of *H. armigera* MCO1 was predicted using the SignalP prediction server (http://www.cbs.dtu.dk/services/SignalP/), and putative transmembrane regions were predicted with TMPred software^21^. GPI anchor sites were predicted with GPI-SOM^22^. MCO1 was subjected to multiple sequence alignments using Clustal X software^23^ and edited with GeneDoc software. The phylogenetic tree was constructed using MEGA4 software^24^.

### Sample preparation

*H. armigera* was collected in different development stages (including eggs, larvae, pupae, and adults). Experiments were performed in three biological replicates, and each biological replicate included at least 15 individuals. The harvested samples were immediately stored at −80 °C for total RNA extraction.

Different tissues (midgut, epidermis, fat body, trachea, hemocytes, salivary gland and Malpighian tubules) were dissected from molting-stage larvae, two-day-old fifth-instar larvae and two-day old adults. Three biological replicates were prepared, and each biological replicate included at least 30 insect tissues. The collected samples were subjected to RNA extraction.

### RNA extraction and cDNA synthesis

The collected *H. armigera* samples were subjected to total RNA extraction using a TRIzol kit (Invitrogen, USA) following the manufacturer’s instructions. The quality and quantity of total RNA were determined by ultraviolet spectrophotometry. Prior to first-strand cDNA synthesis, the total RNA was treated with DNase to prevent genomic DNA contamination. First-strand cDNA was synthesized from 1 μg of total RNA using the PrimeScript RT reagent kit with gDNA Eraser (Takara, Kyoto, Japan) in accordance with the manufacturer’s instructions. The synthesized first-strand cDNA was immediately stored at −80 °C for subsequent use.

### qPCR

The designed primers used in the qPCR analysis are listed in Table S1. The 18S RNA gene in *H. armigera* is stable; therefore, this gene was chosen as a reference gene for normalization in our experiments. qPCR was performed with SYBR Green Supermix (TaKaRa) according to the manufacturer’s instructions. The following qPCR protocol was set: 95 °C for 4 min, followed by 40 cycles of 95 °C for 15 s and 60 °C for 20 s. The specificity of the qPCR signal was further confirmed though agarose gel electrophoresis and melting curve analysis. The comparative Cross Threshold method (CT, the PCR cycle number that crosses the signal threshold) was used to quantify mRNA expression levels^25^.

### dsRNA synthesis

The synthesis of dsRNA was carried out using the MEGAscript RNAi kit (Ambion) following the manufacturer’s instructions. dsRNA templates were obtained by PCR with gene-specific primers containing T7 polymerase sites, as previously described^26^,^27^,^28^. The primer sets are shown in Table S1. PCR amplification was carried out according to the following conditions: 94 °C for 4 min, 35 cycles of 94 °C for 1 min, 60 °C for 1 min and 72 °C for 1 min, and a final elongation at 72 °C for 10 min. The PCR product was further purified and used as a template for *in vitro* dsRNA synthesis. The obtained dsRNA was initially treated with DNase and RNase to remove the template DNA and single-stranded RNA, respectively. The dsRNA was then purified using MEGAclear™ columns (Ambion) and eluted with diethyl pyrocarbonate-treated nuclease-free water. The quality of dsRNA was determined using a biophotometer (Eppendorf). The dsRNA of enhanced green fluorescent protein (EGFP) was used as a negative control.

The effects of RNAi on mRNA expression were analyzed by qPCR. The corresponding primers are shown in Table S1.

### dsRNA injection

In brief, 15 μg of *MCO1* dsRNA was injected into the abdominal intersegment behind the second abdominal segment (one-day-old fifth-instar larvae) using a microinjector with a glass capillary needle. The treated larvae were returned to the artificial diet and reared at 28 °C. Malpighian tubules were dissected at 48 h post-injection to analyze the RNAi efficacy of *MCO1* mRNA by qPCR. Control females were injected with *EGFP* dsRNA.

### Hormone treatment

Fifth-instar larvae (48 h) were chosen to investigate the effect of hormones (JH and 20E) on the expression level of *HaMCO1* transcripts. In brief, 5 μL of 20E (Sigma, USA; 20 ng/μL) or the control solvent was injected into the abdominal legs of larvae. Similarly, 5 μL (8  g/μL) of JH was injected, or 5 μL of the solvent was injected as the corresponding control. Samples were collected at 0.5, 1, 2, 3 and 6 h after 20E or JH injection. At least thirty insects were used in each group, and three biological replicates were prepared. Total RNA was extracted, and subsequent qPCR analysis was performed to investigate the expression level of *MCO1* transcripts.

*ECR*, *USP* and *Met1* dsRNA was generated using similar methods. In brief, 15 μg dsRNA for the 20E receptor (*USP* and *ECR*) and JH receptor (*Met*1) was injected into 24 h fifth-instars larvae, and RNAi efficiency was investigated at different time points (24, 48 and 72 h) by qPCR. At 24 h after dsRNA injection (*USP* dsRNA alone, *ECR* dsRNA alone and *Met1* dsRNA alone), the larvae were further treated with 20E and JH as described above, and samples were further collected at different time points (0.5, 1 and 2 h) after 20E and JH injection. The corresponding solvents of JH or 20E were used as the controls. The expression level of *MCO1* transcript was investigated using qPCR. At least ten insects were used in each group, and three biological replicates were prepared.

### Histological staining of iron in Malpighian tubules cells

The Prussian blue staining method was used to investigate the effect of *MCO1* dsRNA on iron storage in Malpighian tubules^11^. In brief, one-day-old fifth-instar larvae were injected with *MCO1* dsRNA and fed an artificial diet containing 10 mM ferric ammonium citrate for 24 h. Control larvae were injected with *EGFP* dsRNA. After 24 h of maintenance, the treated larvae were fed a regular artificial diet for another 24 h. The Malpighian tubules from the treated larvae were dissected in PBS buffer and fixed in 4% formaldehyde; the fixed tissues were then permeabilized with 1% Tween-20. The Malpighian tubules were incubated with 2% K_4_Fe(CN)_6_ in 0.24 N HCl, rinsed with water and further dissected using an Olympus BX-60 system. The relative fluorescence brightness was determined using Quantity One 4.6.2 software (Bio-Rad). Three biological replicates were prepared, and the corresponding results were compared via Student’s *t*-tests.

### Effects of MCO1 knockdown on transferrin and ferritin

*MCO1* dsRNA (15 μg) was injected into the abdominal intersegment behind the second abdominal segment (one-day-old fifth-instar larvae) using a microinjector with a glass capillary needle. At 48 h, Malpighian tubule samples were dissected, and total RNA was extracted. At least thirty insects were used in each group, and three biological replicates were prepared. The mRNA expression of transferrin and ferritin were investigated using qPCR. Control larvae were injected with *EGFP* dsRNA.

### Statistical analysis

The qPCR experiments were performed in three biological replicates. The results are expressed as the means ± standard deviation (M ± SD) of three biological replicates. The qPCR results were compared using Student’s *t-*tests.

## Additional Information

**How to cite this article**: Liu, X. *et al.* Multicopper oxidase-1 is required for iron homeostasis in Malpighian tubules of *Helicoverpa armigera*. *Sci. Rep.* **5**, 14784; doi: 10.1038/srep14784 (2015).

## Supplementary Material

Supplementary Table S1
