# Supplementary material for: Multicopper oxidase-1 is required for iron homeostasis in Malpighian tubules of Helicoverpa armigera
Source: Sci Rep. 2015 Oct 6;5:14784. doi: 10.1038/srep14784 (PMC4593997; doi:10.1038/srep14784)
Supplement: Supplementary Table S1 [file srep14784-s1.doc]

**Supplementary information**

**Multicopper oxidase-1 is required for iron homeostasis in Malpighian tubules of *Helicoverpa armigera***

Xiaoming Liu^1#^, Chengxian Sun^1#^, Xiaoguang Liu^1^, Xinming Yin^1, 2^, Baohai Wang^3^, Mengfang Du^1^*and Shiheng An^1^*

^1^ State key Laboratory of Wheat and Maize Crop Science/College of Plant Protection, Henan Agricultural University, Zhengzhou 450002 P.R. China

^2^ Department of Agronomy, Xinyang College of Agriculture and Forestry, Xinyang 464000 P.R.China

^3^ Tibet Academy of Agricultural and Animal Husbandry Sciences, Tibet Lhasa 850000 P.R.China

^#^ These authors contribute equally

^*^Correspondence author. Fax: +86 371 63558170.

Email address: anshiheng@aliyun.com (S.-H, An) and [dumengfang@163.com(M.-F](mailto:dumengfang@163.com(M.-F), Du)

**Table S1 List of primers for qPCR and RNAi analysis**

| **Gene** | **Forward primer (5′–3′)** | **Reverse primer (5′–3′)** |
| --- | --- | --- |
| MCO1 | CACAAGCGGAGACAGCACAGG | GGTGTTATTCGGTTTGGGAGC |
| ECR | GGAAGGCTACGAACAACCT | CACTTGAGCACGCCTTTA |
| USP | GCCATCATACTGCTCAACC | CGAGGTGGAAGAAGAACAGAT |
| MET1 | CCATTGACGATGCTCCTG | CACCTTTCGCCTCTTTCT |
| Ferritin | TACCAGGAACAGCCAGCAT | GTCTTGTCCGTGGTTAGCG |
| Transferritin | TGACATCGCTGCTTATTT | GCTGCTAACTTGCCTACT |
| 18S | GCATCTTTCAAATGTCTGC | TACTCATTCCGATTACGAG |
| MCO1-RNAi | GATCACTAATACGACTCACTATAGGGAGATTCCTGAACTGCCCTAT | GATCACTAATACGACTCACTATAGGGAGAGGAACATTCGCAGTATC |
| ECR- RNAi | GATCACTAATACGACTCACTATAGGGAGACGCTGGTATAACAACGGAGGA | GATCACTAATACGACTCACTATAGGGAGAAGCTGGAGACAACTCCTCACG |
| USP- RNAi | GATCACTAATACGACTCACTATAGGGAGACGAACCATCCCCTAAGTGGTTC | GATCACTAATACGACTCACTATAGGGAGACCTTGATGAGCAGGATCTGGTC |
| MET1- RNAi | GATCACTAATACGACTCACTATAGGGAGAGCAACGCAAAGTTGATAAG | GATCACTAATACGACTCACTATAGGGAGATTCTGAATAGGCTGTAGGG |
| EGFP | GATCACTAATACGACTCACTATAGGGAGACCTGAAGTTCATCTGCACCAC | GATCACTAATACGACTCACTATAGGGAGACTCCAGCAGGACCATGTGATC |

18S: 18S rRNA
